# Cross-cultural adaptation and the evaluation of psychometric properties of the Sinhala version of the general rehabilitation adherence scale (GRAS-Sin)

**DOI:** 10.1186/s12891-025-09125-6

**Published:** 2025-09-01

**Authors:** I. Liyanage, D. A. R. K. Dasanayaka, E. Liyanage, H. K. M. Ishara, S. Rathnayake

**Affiliations:** 1 Department of Physiotherapy, Faculty of Allied Health Sciences, University of Peradeniya, Peradeniya, Sri Lanka; 2 Centre for Digital Innovations in Health and Social Care, Faculty of Health Studies, University of Bradford, Bradford, UK

**Keywords:** Cross-cultural adaptation, Physiotherapy treatment adherence, Psychometric properties, Rehabilitation

## Abstract

**Background:**

Physiotherapy adherence can be defined as an active, voluntary patient involvement in a mutually agreed-upon course of action to achieve a desired preventive or therapeutic outcomes. Nonadherence may result in increased healthcare costs and poor recovery. Therefore, it is crucial to measure physiotherapy adherence to eliminate obstacles and promote adherence. No validated tool exists to measure physiotherapy adherence among Sinhala-speaking patients in Sri Lanka.

**Methods:**

This study aimed to develop a Sinhala version of the General Rehabilitation Adherence Scale (GRAS-Sin). During the first phase, the original version of the GRAS was translated and culturally adapted to the Sinhala language. In the second phase, a cross-sectional survey was conducted among 200 patients who sought physiotherapy treatment for musculoskeletal issues at the National Hospital, Kandy and the Teaching Hospital, Peradeniya, Sri Lanka, to evaluate its psychometric properties. The pre-final version of the questionnaire developed in the first phase was used in the second phase.

**Results:**

The majority of the respondents were female (71.5%). Most patients (54.5%) showed a high level (20–24 total scores) of physiotherapy adherence. There was an acceptable level of internal consistency (Cronbach’s alpha = 0.82) and test-retest reliability (intraclass correlation coefficient = 0.907, *p* < 0.05). The content validity index of the scale was 0.89. The sampling adequacy was satisfactory (KMO = 0.755, Bartlett’s test *p* < 0.05). Construct validity was measured by exploratory factor analysis, which revealed a 2-factor model with a 73.4% variance. The incremental fit indices, i.e., the normed fit index, comparative fit index and Tucker-Lewis index, were reported to be > 0.95, whereas the absolute fit index of the root mean square of the approximation error was 0.065. These values indicated a good model fit. No floor and 27% ceiling effects were found. There was a significant (X^2^ = 17.46, *p* = 0.001) relationship between physiotherapy adherence and gender, whereas age, employment status, educational level, and economic status were not significant.

**Conclusion:**

The GRAS-Sin questionnaire is a valid and reliable tool for assessing physiotherapy adherence among Sinhala-speaking patients in Sri Lanka. However, its ceiling effect may limit differentiation at higher adherence levels. Further studies are required to address the limitations of the ceiling effect in the present study.

## Background

Adherence is a complex and intricate phenomenon [1]. Patients who adhere to their healthcare provider’s treatment plan may have better treatment outcomes than those who do not [2]. The term ‘adherence’ is used instead of ‘compliance’ because it implies that patients are actively and voluntarily involved in the planning and implementation of their treatment. On the other hand, ‘compliance’ is seen as simply following the treatment protocol prescribed by the practitioner [3]. In physiotherapy, adherence can be defined as an ‘active, voluntary collaborative involvement of the patient in a mutually acceptable course of behaviour to produce a desired preventative or therapeutic result’ [3].

The physiotherapy treatment consists of both home-based and clinic-based components. Adherence to both home-based and clinic-based components can be measured using questionnaires and patient diaries. The percentage or ratio of attendance is a commonly used measure to assess adherence to the clinic-based component of rehabilitation [4–8].

The degree to which patients do not adhere to physiotherapy treatment remains uncertain [9]. Nonadherence to physiotherapy treatment varies from 14% [10] to 70% [11] and may result in increased healthcare costs and poor recovery. Nonadherence may result from several factors such as personal, disease-related, therapy-related, provider-related, and healthcare system-related factors [1, 3]. It is important to identify these factors [12] and measure physiotherapy adherence to remove barriers and facilitate adherence.

The GRAS is a tool developed and validated to assess the adherence of patients undergoing physical therapy for musculoskeletal issues [12], and it is a tool used to measure the clinic-base component of physiotherapy adherence. It consists of eight questions (asking whether they missed treatment sessions because of some incidents/ events) that should be rated on a Likert scale from 0 to 3, which indicates ‘Always’, ‘Mostly’, ‘Sometimes’, and ‘Never’ respectively. Based on the overall score, physiotherapy adherence is categorised into: high adherence (20–24 points), good adherence (17–19 points), partial adherence (12–16 points), low adherence (8–11 points), and poor adherence (0–7 points) [13]. The tool demonstrates acceptable internal consistency, with a Cronbach’s alpha coefficient of 0.63 and McDonald’s omega (ω) coefficient of 0.77 [12]. Additionally, the test-retest reliability coefficient was found to be 0.88, with a significance level of *p* < 0.01 [12].

Sri Lanka is a middle-income South Asian country that provides rehabilitation services to its population of 20,359,439 individuals [14]. These services are available through both inpatient and outpatient rehabilitation care in secondary and tertiary care institutions in the government sector, as well as in larger private hospitals [15]. To the best of our knowledge, there is no reported validated tool or questionnaire to assess patient adherence to physiotherapy treatment in Sri Lanka. The objective of this study was to culturally adapt the GRAS, initially developed by Naqvi, Hassali [12] in Pakistan, for the Sri Lankan context, and to evaluate the factors that influence adherence to physiotherapy treatment.

## Methods

### Study design

A cross-sectional validation study was conducted in two phases. Phase I involved the cross-cultural adaptation process, while Phase II evaluated the psychometric properties of the GRAS-Sin. Permission for the cross-cultural adaptation of GRAS was obtained from its developers. The guidelines proposed by Beaton et al. [13] for the cross-cultural adaptation process of self-report measures were followed. Factors associated with adherence to physiotherapy treatment were also assessed using the final version of GRAS-Sin. This research was conducted following the Helsinki Declaration. Ethical approval for this study was obtained from the Ethical Review Committee, the Faculty of Allied Health Sciences, University of Peradeniya, Peradeniya, Sri Lanka (No: AHS/ERC/2023/116).

### Phase I: cross-cultural adaptation

Stage one consisted of forward translation, and two bilingual translators who were native speakers of the target language (Sinhala) conducted the initial forward translation. These translations were referred to as T1 and T2, respectively. Stage two consisted of the synthesis of translation. The translators reviewed the T1 and T2 translations by comparing the original questionnaire and created a unified translation, which was termed as T12. This approach enabled the identification of any discrepancies between T1 and T2, and the original version of GRAS. Stage three included Backwards translation. The T12 translation was back-translated by two translators who were proficient in the Sinhala and English languages. This procedure aimed to guarantee that the original content of the T12 version was accurately retained. In stage four, a panel of experts composed of a methodologist, a healthcare professional (Physiotherapist with a Master’s level qualification), a language expert, and a translator meticulously assessed the idiomatic, semantic, experiential, and structural equivalence between the target and the original version. With the exception of question seven, a consensus was reached. In question seven, the term “caregiver” refers to driver, maid and nurse. However, in the Sri Lankan context, informal care is mainly provided by family members, and guardian is the common term used to refer to such family members or carers; therefore, an additional term “guardian” was added. In stage five, pre-testing of the questionnaire was carried out using the pre-final version of GRAS-Sin, involving 20 patients who were seeking physical therapy at the Peradeniya Teaching Hospital, Sri Lanka.

### Phase II: evaluating the psychometric properties of the GRAS-Sin

In Phase II, a cross-sectional survey was conducted to evaluate the psychometric properties of the GRAS-Sin, including reliability (internal consistency and test-retest reliability) and validity (construct validity and content validity) of the GRAS-Sin.

### Sample and study setting

This study was conducted at the outpatient departments of the Teaching Hospital, Peradeniya and the National Hospital, Kandy, Sri Lanka, with the approval of the directors of the respective hospitals. A minimum of 200 subjects is required for exploratory factor analysis to obtain factor solutions [16]. The GRAS consists of eight items. The suggested item-to-response ratio range from 1:3 to 1:20 [17]. Therefore, a sample of 200 Sinhala-speaking patients who attended physiotherapy treatment at both hospitals was selected via systematic random sampling to serve as the sample for the psychometric evaluation. Data collection was continued until the required sample size was met. Patients with musculoskeletal problems who had undergone physical therapy for at least 2 weeks were included in the study. Patients who were seeking treatment for problems other than musculoskeletal problems and patients who were younger than 18 years were excluded from the study. In the test-retest reliability assessment, 20 patients participated.

### Study instrument

Data were collected through a self-administered questionnaire, which consisted of pre-final versions of the GRAS-Sin and questions related to socio-demographic data, including age, education level, income, occupation, medical, and physiotherapy diagnosis. Data collection took place from February to May 2024, with a retest conducted at a 3-week interval.

### Statistical analysis

Demographic information and physiotherapy adherence data were presented as frequencies, percentages, means and standard deviations. The relationships between physiotherapy adherence and patient characteristics were assessed via the chi-square test. All the analyses were conducted using SPSS version 25, and significant tests were conducted at a 5% significance level with a 95% confidence interval. The reliability, in terms of internal consistency and test-retest reliability, was assessed. The internal consistency was estimated by calculating Cronbach’s alpha coefficient for the scale and the subscales [18]. Cronbach’s alpha value of 0.70 or higher was considered acceptable [19]. Intra-class correlation coefficiency (ICC) one-way random-effects model was used for the analysis of test-retest reliability [20], in which values < 0.40 were considered as poor agreement, 0.40 to 0.75 as fair-to-good agreement, and > 0.75 as excellent agreement [21]. The content validity index (CVI) was calculated to assess the agreement of the subject experts to keep the eight questions in the questionnaire. Seven reviewers assessed the data. A value of 0.8 or higher was considered acceptable [22]. Floor and ceiling effects were calculated to find extreme items that reduced the content validity of the questionnaire. Less than 15% of the floor or ceiling effect can be considered as good content validity [23]. The construct validity was assessed using exploratory factor analysis through Principal Component Analysis (PCA). The factorability was assessed by examining the correlation matrix, Kaiser–Meyer–Olkin measure of sampling adequacy and Bartlett’s test of sphericity. The model was confirmed through partial confirmatory factor analysis (PCFA) via maximum likelihood (MLA) with Oblimin rotation. The model developed using PCA and PCFA was presented with the null model chi-square and implied model chi-square values and their degrees of freedom. The incremental fit indices; normed fit index (NFI), comparative fit index (CFI) and Tucker Lewis index (TLI) were used for good model fit if the values are > 0.95, which were calculated using null model chi-square and implied model chi-square values [24].

## Results

### Patient characteristics and physiotherapy adherence

**Fig. 1:**
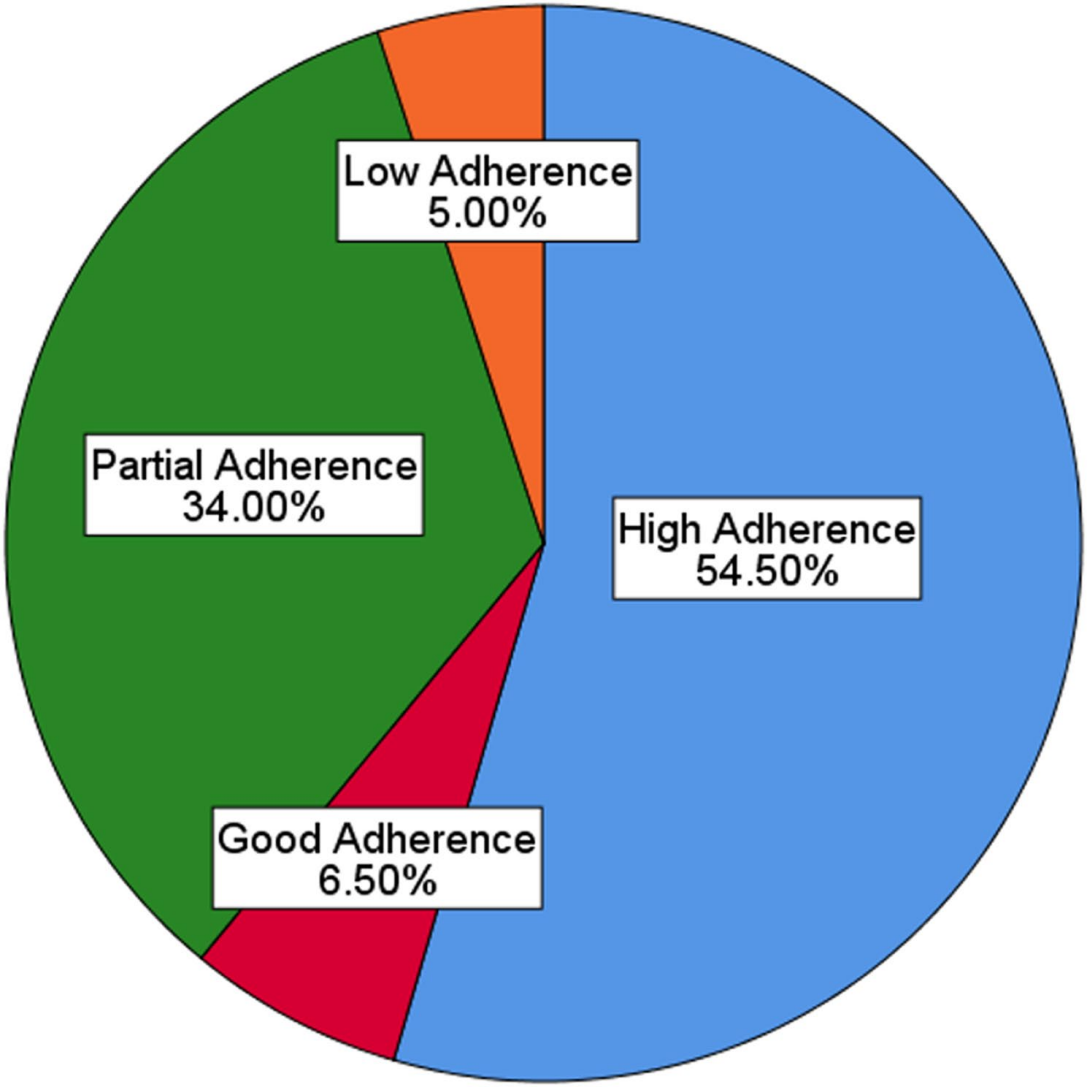
Treatment adherence

Two hundred patients responded, where age of the participants ranged from 18 to 85 years, and the majority were aged between 56 and 65 years (33%). The majority of participants were females (71.5%) and had education above the primary level (85.5%). From the sample, most were unemployed (46.5%) and had middle income (54.5%) (Table 1). All study participants suffered from different musculoskeletal conditions where, 47 (23.5%) of them presented with knee osteoarthritis, 50 (25%) with lower back pain, 32 (16%) with fractures in the upper and lower limbs, 26 (13%) with shoulder joint pathologies and others with soft tissue injuries and dislocations of different joints in minor propositions 45( 22.5% ). Only 65 of them had noncommunicable diseases; 19 (29%) had diabetes, and 17 (26%) had hypertension, which may affect the musculoskeletal condition of the individuals. All these participants were receiving physiotherapy rehabilitation and pharmacotherapy. The therapeutic interventions that they received vary with the disease condition and include electrical modality treatments, joint mobilisation and soft tissue mobilisation. Among the participants, 54.5% had a high level of physiotherapy adherence (Fig. 1). Treatment adherence showed no significant association with the participants’ respective disabilities or dysfunctions.

**Table 1:** Descriptive statistics (*n* = 200)

Sociodemographic information		Frequency (*n*)	Percentage (%)
Age	18-25 yrs	15	7.5
	26-35 yrs	10	5.0
	36-45 yrs	23	11.5
	46-55 yrs	45	22.5
	56-65 yrs	66	33.0
	66-75 yrs	34	17.0
	76-85 yrs	7	3.5
Gender	Male	57	28.5
	Female	143	71.5
Education level	No formal education	2	1.0
	Primary	25	12.5
	Ordinary level	86	43.0
	Advanced level	71	35.5
	Tertiary	14	7.0
	Missing	2	1.0
Employment sate	Government	19	9.5
	Private	46	23.0
	Self-employed	9	4.5
	Retired	33	16.5
	Unemployed	93	46.5
Income	Low income	91	45.5
	Middle income	109	54.5
	High income	0	0.0
Presented musculoskeletal conditions	Knee osteoarthritis	47	23.5
	Lower back pain	50	25
	Upper and lower limb fractures	32	16
	Shoulder joint pathologies	26	13
	Soft tissue injuries, dislocations and others	45	22.5

The associations between treatment adherence and sociodemographic information were measured via the chi-squared test, and only gender was significantly associated with treatment adherence, with females being more adherent than males (Table 2).

**Table 2:** Associated factors of treatment adherence (*n* = 200)

Sociodemographic factors	Chi-square value	Sig. *p* value
Age	15.897	0.60
Gender	17.463	0.001^*^
Education level	15.744	0.203
Employment state	18.247	0.108
Income	2.775	0.428

*Significant p values, at a 5% significance level and 95% confidence interval

### Reliability

Internal consistency (Cronbach’s alpha = 0.82) was deemed acceptable, and the test-retest reliability (ICC = 0.907, *p* < 0.05) fell within an excellent range.

### Content validity

The CVI of the 8-item scale was 0.89, calculated as the average of the item content validity index (I-CVI) of each of the eight items, and it was decided to include all the questions in the questionnaire (Table 3). No floor and 27% ceiling effects were found for the total score of the questionnaire.

### Construct validity

PCA with Varimax rotation revealed a 3-factor model (eigenvalues > 1) with a 73.4% variance. The sampling adequacy was satisfactory (KMO = 0.755, Bartlett’s test p-value < 0.05). Factor 1 accounted for 44.5% of the total variance, and factors 2 and 3 accounted for 15.2% and 13.6% of the variance, respectively. Factor 1 was named as ‘patient-related factors’. Based on the scree plot, as only one item remained within factor 3, factor 2 and factor 3 were combined and named as ‘accessibility’ (Table 3).

**Fig. 2:**
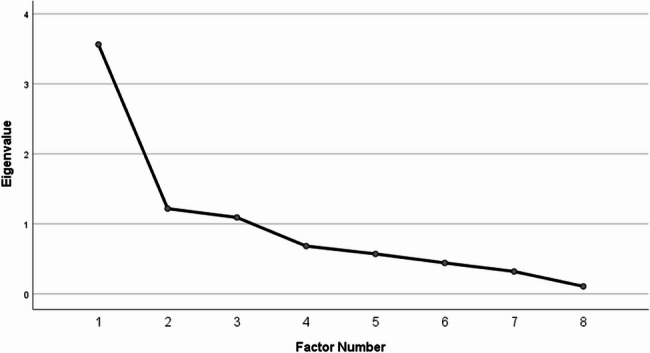
Scree plot of the two-factor model confirmed through the PCFA via MLA with Oblimin rotation

This two-factor model was confirmed through PCFA using MLA with Oblimin rotation (Fig. 2). The KMO value was 0.755, with a significant Bartlett’s test p-value < 0.05. The null model chi-square and implied model chi-square values were reported as 694.454 and 12.898, respectively. The incremental fit indices, NFI, CFI and TLI, were reported as 0.98, 0.99 and 0.96, respectively whereas the absolute fit index of the root mean square of the error of approximation (RMSEA) was 0.065. These values indicated a good model fit.

**Table 3:** I-CVI and factor structure

Item number	Item	I-CVI	Component	
			1	2
1	Other commitments	1	0.891	
2	Unable to manage time	1	0.918	
3	Feel well	1	0.857	
4	Excessive pain	1	0.477	
7	Unavailability of the guardian	0.71	0.481	
5	Treatment cost	0.86		0.621
6	Not worth the money spent	0.71		0.900
8	Unavailability of a therapist	0.86		0.939

## Discussion

This study is the first to adapt GRAS cross-culturally and to assess its psychometric properties for measuring physiotherapy adherence among Sinhala-speaking patients in Sri Lanka. The GRAS-Sin was adapted from the original GRAS to the Sinhala language and had acceptable content validity, construct validity, and reliability, including test-retest reliability and internal consistency. Consequently, this instrument is suitable for evaluating physiotherapy treatment adherence among Sinhala-speaking patients.

It is important to measure physiotherapy adherence to identify barriers and overcome them. Several scales have been developed to assess physiotherapy adherence, namely the Exercise Adherence Scale (ERAS) [25] and the Adherence to Exercise for Musculoskeletal Pain Tool (ATEMPT) [26]. However, the GRAS has been developed to assess physiotherapy adherence among patients seeking treatment for musculoskeletal problems in Pakistan. Therefore, this tool was chosen for cross-cultural adaptation as similarities could be identified between Pakistan and Sri Lanka, as both countries are located in the South Asian Region. Cross-cultural adaptation is required when a tool is adapted to a different setting. The process was completed in five stages [13]. The translation process was carried out without major difficulties except for question seven. The original questionnaires have been altered in previous cross-cultural studies [27, 28]. Hence, for question number seven, the Sinhala word “BARAKARU,” which means guardian, was added to adapt to Sri Lankan culture.

The GRAS-Sin reported good Cronbach’s alpha values, which were similar to those of the original version of the questionnaire, indicating good internal consistency. In accordance with Fleiss [29], the ICC (test-retest reliability) is categorised as follows: less than 0.40 is considered poor, 0.40–0.75 indicates fair to good reliability, and greater than 0.75 signifies excellent reliability. Therefore, the test-retest reliability of the instrument shows excellent consistency across tests. The original development study yielded similar results [12]. These findings suggest that the GRAS-Sin is a reliable tool for evaluating physiotherapy treatment adherence in the Sinhalese-speaking population in Sri Lanka.

The original version of the questionnaire had a CVI of 0.89 [12], and cross-culturally adapted versions also demonstrated similar values, indicating good content validity. The construct validity of the GRAS-Sin was evaluated by EFA and PCA with varimax rotation. A recent systematic review and meta-analysis emphasised the importance of verifying sample size adequacy via multiple measures for a widely used questionnaire tool [30]. The sampling adequacy was satisfactory. PCA revealed a 3-factor model that was fixed and confirmed as a 2-factor model. The incremental fit indices, i.e., NFI, CFI and TLI, were reported to be > 0.95. In contrast, the absolute fit index of the root mean square of the error of approximation showed a good fit model. Similar results were observed in the original development of the questionnaire [12]. The original GRAS scale demonstrated a total variance of 62.3; however, the GRAS-Sin demonstrated a total variance of 73.4, indicating a better fit than the original version. The factor loading for the factors was slightly different from the original development. In the original version, question number seven was loaded with factors related to accessibility, but in the adaptation for the Sri Lankan context, it was loaded with personal factors. This change may be due to the alteration of the question, which included a Sinhala word, making the question personal.

Various factors can influence adherence to physiotherapy treatment, and these factors can differ across studies.Essery et al. [31] reported that the intention to engage in home-based physical therapies, self-motivation, self-efficacy, previous adherence to exercise-related behaviours, and social support influence adherence. In this study, only gender was found to be associated with treatment adherence among demographic factors. However, it is worth noting that some studies have reported that not all demographic factors are linked to adherence [1, 32–34]. Additionally, a systematic review has indicated that the impact of education on adherence is still uncertain [35].

The majority of patients in this study had high adherence to physiotherapy treatment. However, contradictory results have been reported, indicating that most patients do not adhere to physiotherapy treatment in Pakistan [12]. This difference may be attributed to factors such as patient satisfaction and free physiotherapy treatment offered by public sector hospitals in Sri Lanka, where patients need to cover only transport costs, as the treatment cost is the main barrier to rehabilitation [36].

### Limitations of the study

This study was carried out at government hospitals, which provide 90% of the inpatient care and 50% of the outpatient care in Sri Lanka. Therefore, the physiotherapy adherence in this study may not accurately represent the treatment adherence of patients attending private-sector hospitals in Sri Lanka, which is one drawback of the study. One limitation of the tool is the ceiling effect, which diminishes its ability to effectively distinguish patients with high reported adherence. To address this issue, the supplementary application of a more subtle instrument may be necessary to achieve accurate differentiation within the high-adherence population. Future research should prioritise refining the tool to enhance its ability to identify patients with high adherence.

## Conclusion

The GRAS-Sin questionnaire demonstrates validity and reliability in assessing physiotherapy adherence among Sinhala-speaking patients following physiotherapy treatment for musculoskeletal problems in Sri Lanka. However, the presence of a ceiling effect may limit its ability to differentiate adherence levels at the upper range. To address this limitation, a more subtle instrument may be required for accurate differentiation. Further studies are required to address the limitations of the ceiling effect in the present study, ensuring a more comprehensive assessment of adherence variations among Sinhala-speaking patients. The majority of patients seeking physiotherapy treatment for musculoskeletal issues show high adherence to physiotherapy treatment.

## Data Availability

The datasets used and/or analysed during the current study are available from the corresponding author upon reasonable request.

## Abbreviations

ATEMPT: Adherence to exercise for musculoskeletal pain tool
CFI: Comparative Fit Index
CVI: Content Validity Index
EFA: Exploratory factor analysis
ERAS: Exercise Adherence Scale
GRAS: General Rehabilitation Adherence Scale
GRAS-Sin: Sinhalese version of the General Rehabilitation Adherence Scale
ICC: Intraclass correlation coefficient
I-CVI: Item Content Validity Index
MLA: Maximum likelihood
NFI: Normed Fit Index
PCA: Principal component analysis
PCFA: Partial confirmatory factor analysis
TLI: Tucker Lewis Index
